# The influence of college students' academic stressors on mental health during COVID-19: The mediating effect of social support, social well-being, and self-identity

**DOI:** 10.3389/fpubh.2022.917581

**Published:** 2022-09-20

**Authors:** Peng Li, Jiaqi Yang, Zhao Zhou, Zijing Zhao, Tour Liu

**Affiliations:** ^1^Key Research Base of Humanities and Social Sciences of the Ministry of Education, Academy of Psychology and Behavior, Tianjin Normal University, Tianjin, China; ^2^Faculty of Psychology, Tianjin Normal University, Tianjin, China; ^3^Collaborative Innovation Center of Assessment Toward Basic Education Quality, Beijing Normal University, Beijing, China; ^4^Tianjin Social Science Laboratory of Students' Mental Development and Learning, Tianjin Normal University, Tianjin, China

**Keywords:** academic stressors, mental health, social support, social well-being, self-identity, work stressor

## Abstract

COVID-19 caused harmful mental consequences to the public, and mental health problems were very common among college students during the outbreak of COVID-19. Academic stressors were the main stress for college students, and social support, social well-being, and self-identity were widely known as protective factors for mental health. Therefore, the study aimed to investigate the influence of academic stressors on mental health and the mediating effect of social support, social well-being, and self-identity among college students during the outbreak of COVID-19. With 900 college students as subjects, using the college students' academic stressors questionnaire, social support questionnaire, social well-being scale, self-identity scale, and depression anxiety stress scales (DASS-21), the results showed that: (1) academic stressors had a significantly negative correlation with social support, social well-being, and self-identity while having a significantly positive correlation with mental health; (2) academic stressors could positively predict mental health; (3) this effect was mediated by social support, social well-being, and self-identity; (4) work stressor was an important stressor during COVID-19, and had the same role as academic stressors in the structural equation model. The results of this study suggested that adjusting the academic stressors or work stressors of college students and enhancing social support could improve social well-being and self-identity, and might effectively protect their mental health under the COVID-19 pandemic environment.

## Introduction

College was an important turning point and the critical period for individuals from adolescence to adulthood in psychology. Mental health problems were very common in this period (1). A large number of mental state investigations showed that COVID-19 spoke common mental health issues, such as stress, anxiety, and so on (2–5). As COVID-19 broke out, it swept across many countries and even the world, causing damaging effects on the mental health of the public (6). For college students, the accompanying results of a pandemic like lockdown and taking online courses had already led to negative consequences and more stressors (7–9). Most existing studies focused on mental health for general populations (10) or health care workers (11), whose results might not be applicable to college students. So it was important and necessary to explore the protective factors in the relationship between stressors and mental health under the context of COVID-19 among college students.

## The mental health of college students and their academic stressors

Various mental health challenges ranging from excessive stress and anxiety to severe depression had emerged (12). Cumulative stressors were the cause of anxiety, depression, and mental problems (13, 14). Anxiety was a common kind of negative emotion generated when faced with unpleasant events or a challenge that was too difficult to deal with (15). After COVID-19, the ratio increased up to 24.9% (16). Depression produced severe persistent sadness, unhappiness, mood swings, behavior, and mental disorders (17). Everyone lived with depression and the prevalence was even higher (18).

It was known that stressors or pressure were associated with individuals' mental health (19–21). Researchers proved that academic stressors had the main pressure on college students (22, 23). They would experience a variety of academic stressors on campus, such as giving a class presentation, solving problems against the clock, and dealing with tests and examinations. Those stressors aroused in an educational environment were called academic stressors (24). According to previous studies, academic stressors were associated with the rise of anxiety and depression (25–27) and had a positive correlation with mental health (28, 29).

## The effects of the COVID-19 pandemic on mental health and academic stressors of college students

COVID-19 as a natural disaster could have strong effects on individuals' mental health (30, 31). It swept across the world, causing damaging effects on the mental health of the public (6). The pandemic embodied many overwhelming stressors, such as losing employment, financial insecurity, and isolation from others (32), increasing adverse psychological consequences on individuals, like depression (7, 8). There was reason to be concerned about the rapid and possibly sustained negative impact of the COVID-19 pandemic on mental health (33). College students were an important group who was in the process to get into society. In a recent study, approximately two-thirds of participants reported anxiety and depression symptoms in the moderate to severe range, and about one-third reported suicidality (34). The accompanying results of the pandemic, such as lockdown and taking online courses, led to negative consequences and more stressors for college students (7–9). Many students reported high academic stressors (35, 36), and this situation was even more severe during the pandemic. Home-study initiatives caused disruption of course in person and uncertainty of back to campus, which made college students experience poor mental health (37). Also, research showed that disruptions of research projects and internships would jeopardize the process of study, delay graduation, and undermine competitiveness in the job market, which in turn fuel anxiety among college students (9).

## The role of social support, social well-being, and self-identity

Social support was the general or specific social resources that an individual obtained from others that could help to cope with difficulties and crises in life (38). As mental problems are widely encountered among college students, it was important to give them support from family, university, or friends (39), which was beneficial for reducing mental problems during COVID-19 (40). The negative correlation between academic stressors and social support reflected that someone who had lower support would suffer more academic stressors (41, 42). Social support had been long established as a mediator in buffering the impact of stressors (41, 43–46), and as a protective psychological factor, it would reduce the effect of academic stressors on mental health (47). Therefore, hypothesis 1 was put forward: Academic stressors directly positively predicted mental health, and social support played an indirect mediating role.

Social well-being referred to the individual's feelings toward the quality of relationships between himself, others, societies, and also self-assessment of their living environment and social functioning (48, 49). Research showed that social support played a significantly positive predictor of social well-being (44), which meant that if an individual received enough support from families, friends, or any others, they would have a higher level of well-being. In a time when students faced uncertainty and a continually changing environment, COVID-19 lowered individuals' well-being (50). Research showed that academic stressors were significantly negatively correlated with well-being, and social support mediated the relationship (42, 51–53). Thus, hypothesis 2 was put forward: Academic stressors directly negatively predicted social well-being, and social support played an indirect mediating role.

Self-identity was proposed by Erikson (54) as the maturity, continuity, and integration of individual personality development, mainly formed in youth. Previous research on social cognition suggested that emphasizing self-identity was key to changing a person's behavior and it would be effective in changing intentions and behaviors under the situation of the COVID-19 outbreak (55). Palsane (56) concluded that self-incongruence was related to a high level of stress, and poor physical and mental health. A common assertion about the relationship between social support and self-identity was that social support provided reassurance to the individual that esteemed and valued; that boosted the individual to believe that he could cope with or adjust to life's exigencies (43, 57, 58). In addition, many studies provided evidence that social support and well-being were associated with mental health benefits (59, 60). Therefore, it was reasonable to propose hypothesis 3: Social support negatively predicted mental health with social well-being and self-identity mediating the effect.

## The chain mediating effect of social support, social well-being, and self-identity

According to the Stress-Buffering theory, the negative impact of stressors exposure was weakened when there were enough coping resources. Previous studies had demonstrated that social support and self-esteem were coping resources to reduce stressors (61). Receiving social support, such as care, attention, and being valued by others could serve as an effective buffer to challenging life events and enhance mental well-being (62). And self-identity had important self-aspects as self-esteem (63), which might have a stress-buffering role. Therefore, social support, social well-being, and self-identity all could be considered as stress-buffer factors and had positive functions to protect mental health (41, 43–46, 50, 55, 59, 60). Hong et al. (64) pointed out that related stressors in life would first affect individual emotional response and attitude toward life, and then ultimately affect mental health through positive or negative emotions as suggested. As there were few studies about stress-buffering effects on mental health, so it was of great value to consider social support, social well-being, and self-identity, underlying the following study and specific measurements. It was reasonable to give the hypothesis that social support, social well-being, and self-identity had a chain mediation effect.

## The purpose of the present study

**Figure 1:**
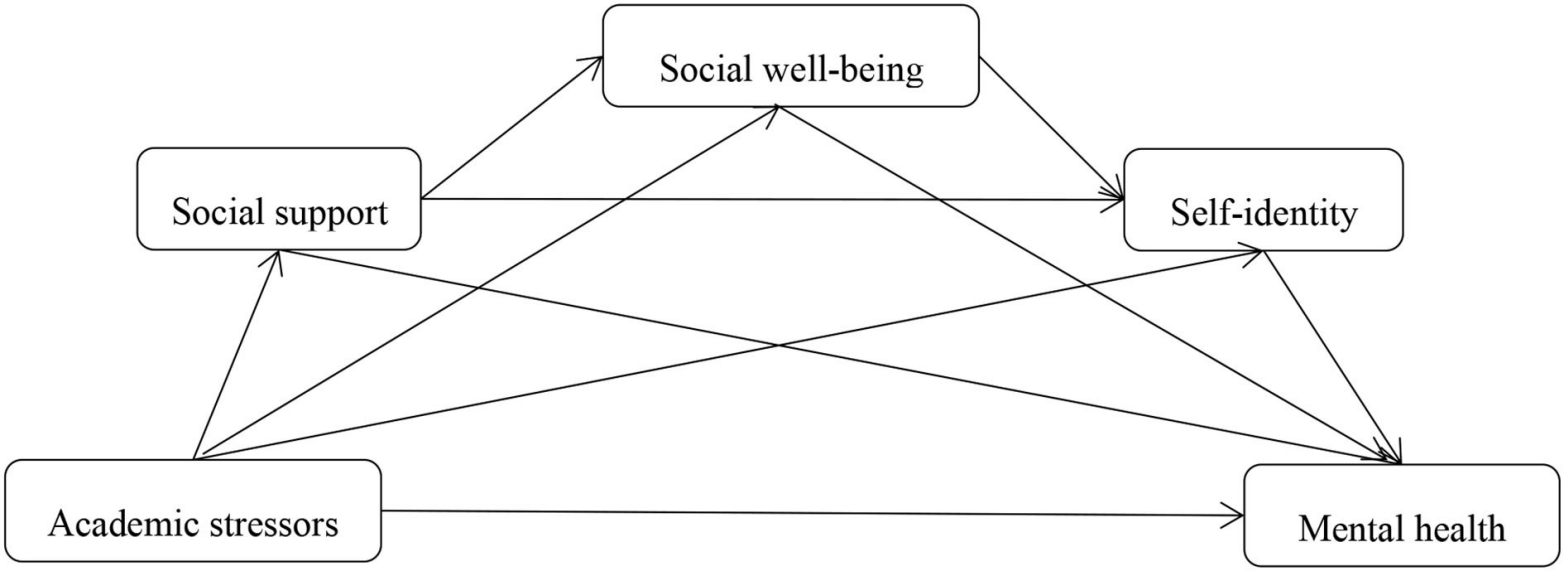
The hypothesis of mediating effect of academic stressors on mental health of college students.

Combining hypothesis 1, hypothesis 2, and hypothesis 3, a model consisting of academic stressors, social support, social well-being, self-identity, and mental health was conducted, in which academic stressors had a direct influence on mental health, social support, social well-being, and self-identity playing as mediators under the COVID-19 environment. Specifically, mental health was negatively associated with social support, social well-being, and self-identity, while positively correlated with academic stressors. In addition, social support, social well-being, and self-identity were negatively associated with academic stressors. And those three variables positively correlated with each other. See Figure 1 for details.

## Materials and methods

### Participants

The survey began in July 2020, about 6 months after the first outbreak of COVID-19. By distributing online questionnaires among Chinese college students, a total of 900 participants were collected using random sampling. There were 900 valid questionnaires with no missing data. Participants in this study consisted of 290 male students and 610 female students. There were 588 (65.33%) undergraduate, 268 (29.78%) master students, and 44 (4.89%) doctoral students. Among them 34 (3.78%) were in Grade one, 136 (15.11%) in Grade Two, 224 (24.89%) in Grade Three, and 506 (56.22%) in Grade Four. The average age of the participants was 21.95 with a standard deviation of 3.36.

### Ethics statement

The study involving human participants was reviewed and approved. All procedures performed in studies involving human participants were in accordance with the ethical standards of the Academic Board of Tianjin Normal University and with the 1964 Helsinki declaration and its later amendments or comparable ethical standards. The participants voluntarily completed the questionnaire. All were asked to sign a consent form.

### Measures

#### College students' academic stressors questionnaire

The academic stressors of college students were measured by the College students' academic stressors questionnaire revised by Chen (65). The questionnaire consisted of 49 questions in 8 factors (work stressor, goal stressor, task stressor, competitive stressor, obstacle stressor, parental stressor, others' expectation stressor, and environmental stressor). Each item was scored from 1 (no stress) to 5 (high stress). The higher the total score was, the more academic stressors suffered. The reliability of the questionnaire in this study was good (Cronbach's α = 0.973). The structural validity of the questionnaire in this study was acceptable (*RMSEA* = 0.056, *CFI* = 0.906, *TLI* = 0.896, *SRMR* = 0.044).

#### Social support questionnaire

The social support questionnaire was developed by Xiao (66). There were 10 questions in total, which were divided into three dimensions: subjective support, objective support, and utilization degree of support. In the questionnaire, items 1–4 and items 8–10 were scored between 1 and 4. Item 5 had four options, each of which was scored from 1 (none support) to 4 (full support). Items 6 and 7 were designed to investigate the sources of support received, scoring a point for each item selected. The total score was calculated as the social support score. The higher the score, the better the situation of social support. The Cronbach's alpha of the questionnaire in this study was 0.814. The structural validity of the questionnaire in this study was good (*RMSEA* = 0.054, *CFI* = 0.910, *TLI* = 0.901, *SRMR* = 0.042).

#### Social well-being scale

The social well-being scale, revised by Miao y Wang (67), consisted of 15 questions and was divided into five dimensions: social acceptance, social actualization, social integration, social coherence, and social contribution. A 7-point score was adopted, with the score from obviously not applicable at all to obviously very applicable being 1–7, respectively. The higher the score, the better the situation of social well-being. In this study, the Cronbach's alpha of the scale was 0.847. The structural validity of the scale in this study was good (*RMSEA* = 0.069, *CFI* = 0.963, *TLI* = 0.952, *SRMR* = 0.045).

#### Self-identity scale

The self-identity scale was developed by Oakes y Prager based on Erickson's theory (68). The scale consisted of 19 items, scoring from 1 (not applicable at all) to 4 (very applicable). After the reverse score of the reverse questions, the total score of the questionnaire was calculated. The higher the score was, the better the self-identity. The Cronbach's alpha coefficient of this scale in this study was 0.833. The structural validity of the scale in this study was good (*RMSEA* = 0.056, *CFI* = 0.918, *TLI* = 0.901, *SRMR* = 0.054).

#### Depression anxiety stress scales (DASS-21)

Mental health was measured by the simplified version of self-assessment lists of depression–anxiety–pressure (Depression Anxiety Stress Scales, DASS). DASS first was put forward by P. F. Lovibond y S. H. Lovibond (69). This research adopted the DASS-21 scale assessment survey participants 1 week before the survey of psychological state, with each participant scoring between 0 and 3 points: “0” for “never,” “1” means “sometimes,” “2” means “often,” and “3” means “always.” The sum of the scores was multiplied by 2 as the final score. The Cronbach's alpha coefficient was 0.939 in this study. The structural validity of the scale in this study was good (*RMSEA* = 0.071, *CFI* = 0.916, *TLI* = 0.903, *SRMR* = 0.040).

### Data analysis

Harman's one-factor test was used to analyze the common method bias. The results showed that the cumulative interpretation of the first factor was 19.49, <50%, suggesting that there was no common method bias (70). SPSS 24.0 was used to conduct Pearson correlations, and Mplus 7.0 was used for mediation analysis.

## Results

### Descriptive statistics

The descriptive statistics of other variables are shown in Table 1. The statistical values of skewness and kurtosis of the variables in this research were normal distribution. The mean of academic stressors was 137.30 and the standard deviation was 34.32 in this study, much higher than the criterion provided by Chen (65). The corresponding mean and standard deviation values in Chen (65) were 131.76 and 31.77, respectively. *T*-test was conducted using the mean and standard deviation from the original article with the results in this study. A significant difference was found (*t* = 3.31, *p* < 0.05), suggesting COVID-19 did cause high academic stressors among college students.

**Table 1:** Descriptive statistics for main variables.

**Variables**	**Minimum**	**Maximum**	**Mean**	**SD**	**Skewness**	**Kurtosis**
Academic stressors	49.00	245.00	137.30	34.32	−0.19	−0.06
Social support	22.00	58.00	39.06	6.30	−0.03	−0.27
Social well-being	21.00	105.00	75.40	13.89	−0.26	0.01
Self-identity	30.00	72.00	53.61	6.89	−0.23	0.13
Mental health	42.00	168.00	64.08	20.28	1.33	2.06

### Correlation analysis

The correlation analysis for academic stressors, social support, social well-being, self-identity, and mental health was conducted in this study. Results in Table 2 showed that academic stressors were positively correlated with mental health (*r* = 0.40). And academic stressors was negatively correlated with social support (*r* = −0.20), social well-being (*r* = −0.24), and self-identity (*r* = −0.44). In addition, social support, social well-being, and self-identity were positively correlated with each other (*r* = 0.46–0.54). Furthermore, there were eight different stressors in academic stressors: work stressor, goal stressor, task stressor, competitive stressor, obstacle stressor, parental stressor, others' expectation stressor, and environmental stressor. To investigate their roles, a partial correlation was conducted. The partial correlation results are shown in Table 3. It could be seen that only the work stressor was significantly correlated with social support, social well-being, self-identity, and mental health, after controlling the other seven stressors.

**Table 2:** Correlation analysis results of all variables.

**Variables**	**1**	**2**	**3**	**4**	**5**
1 Academic stressors	1				
2 Social support	−0.20***	1			
3 Social well-being	−0.24***	0.46***	1		
4 Self-identity	−0.44***	0.46***	0.54***	1	
5 Mental health	0.40***	−0.38***	−0.43***	−0.69***	1
*M* ±*SD*	137.30 ± 34.32	39.06 ± 6.30	75.40 ± 13.89	53.61 ± 6.89	64.08 ± 20.28

^***^p < 0.001.

**Table 3:** Partial correlation analysis results.

**Variables**	**Social support**	**Social well-being**	**Self-identity**	**Mental health**
Work stressor	−0.116***	−0.122***	−0.165***	0.138***
Goal stressor	−0.037	0.048	0.016	−0.011
Task stressor	0.006	−0.022	0.065	−0.036
Competitive stressor	−0.027	−0.071*	0.025	−0.087**
Obstacle stressor	−0.023	−0.007	0.022	−0.082*
Parental stressor	−0.020	−0.012	−0.009	0.002
Others' expectation stressor	−0.320	−0.098**	0.058	−0.015
Environmental stressor	0.130***	0.144***	−0.044	0.113***

^*^p < 0.05,

^**^p < 0.01,

^***^p < 0.001.

### Mediation model results

**Figure 2:**
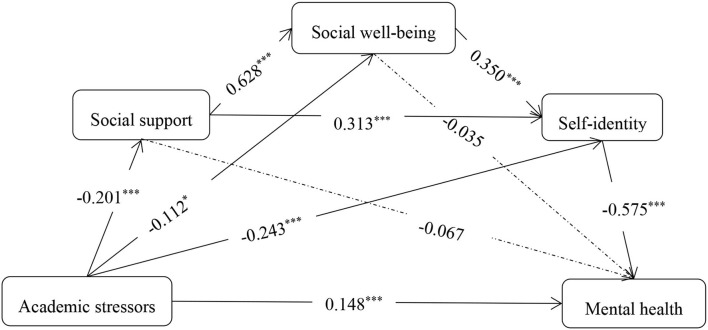
The mediation model of college students' academic stressors on mental health. *p < 0.05, ***p < 0.001.

Take academic stressors as the independent variable, social support, social well-being, and self-identity as mediation variables to conduct four structural equation models (SEM), because there were depression, anxiety, and stress scores included in DASS-21 (71, 72). So four SEMs results are shown in Table 4, with depression, anxiety, stress, and mental health as the dependent variable, respectively. It could be known that the model fit results of mental health were slightly greater than the other three, and Zanon et al. (73) suggested that the DASS-21 could be used as a unidimensional scale, so the total score of DASS-21 was used in the following analysis. The path coefficients are shown in Figure 2, and the corresponding intermediatory effects and their confidence intervals of the model are shown in Table 5.

**Table 4:** Model fit results of the four SEMs.

**Dependent variables**	***RMSEA***	***CFI***	***TLI***	***SRMR***
Depression	0.070	0.909	0.884	0.038
Anxiety	0.071	0.907	0.882	0.039
Stress	0.070	0.910	0.885	0.038
Mental health	0.066	0.920	0.901	0.038

**Table 5:** The chain mediating effect of social support, social well-being and self-identity between academic stressors and mental health.

**Path**	**Effect**	**Boot SE**	**Boot LLCI**	**Boot ULCI**
Direct effect	0.148	0.027	0.098	0.197
Academic stressors → self-identity → mental health	0.140	0.020	0.104	0.184
Academic stressors → social well-being → mental health	0.004	0.006	−0.006	0.018
Academic stressors → social support → mental health	0.013	0.014	−0.015	0.046
Academic stressors → social well-being → self-identity → mental health	0.023	0.009	0.007	0.042
Academic stressors → social support → self-identity → mental health	0.036	0.010	0.020	0.062
Academic stressors → social support → social well-being → mental health	0.004	0.007	−0.009	0.018
Academic stressors → social support → social well-being → self-identity → mental health	0.025	0.006	0.015	0.040
Total mediating effect	0.246	0.025	0.200	0.297
Total effect	0.394	0.030	0.328	0.448

As shown in Table 5, the confidence intervals of the three paths: academic stressors → social well-being → mental health, academic stressors → social support → mental health, academic stressors → social support → social well-being → mental health included 0, so the mediating effects were not significant. Instead, confidence intervals of four paths: academic stressors → self-identity → mental health, academic stressors → social well-being → self-identity → mental health, academic stressors → social support → self-identity → mental health, academic stressors → social support → social well-being → self-identity → mental health did not include 0, so the mediation effects were significant. The total mediating effect accounted for 62.44% of the total effect. The simple mediating effect of self-identity accounted for 56.91% of the total mediating effects. Generally, the results suggested that social support, social well-being, and self-identity could mediate the impact that academic stressors on mental health, and self-identity was the most important mediation variable.

**Figure 3:**
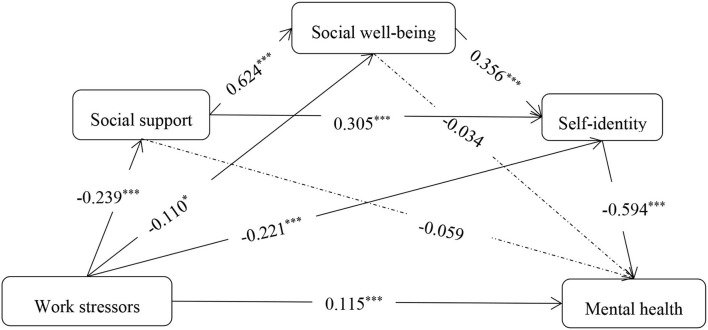
The mediation model of college students' work stressors on mental health. *p < 0.05, ***p < 0.001.

It could be known from the partial correlation results that work stressor was the most important dimension. To explore the work stressors subdimension of academic stressors, introducing it as the independent variable, mental health as the dependent variable, social support, social well-being, and self-identity as intermediatory variables, conduct the mediation analysis. Model fit results: *RMSEA* = 0.055, *CFI* = 0.953, *TLI* = 0.931, *SRMR* = 0.031. The corresponding intermediatory effects and confidence intervals of the model are shown in Table 6, and the corresponding mediation figure is Figure 3. The total mediating effect accounted for 68.66% of the total effect. The simple mediating effect of self-identity accounted for 51.98% of the total mediating effects. The SEM in which work stressor was introduced as the independent variable and the SEM in which academic stressors was considered as the independent variable had significant paths, suggesting social support, social well-being, and self-identity could mediate the impact that work stressors had on mental health. Work stressor was the important factor during COVID-19 among academic stressors.

**Table 6:** The chain mediating effect of social support, social well-being and self-identity between work stressors and mental health.

**Path**	**Effect**	**Boot SE**	**Boot LLCI**	**Boot ULCI**
Direct effect	0.115	0.027	0.063	0.166
Work stressors → self-identity → mental health	0.131	0.019	0.097	0.172
Work stressors → social well-being → mental health	0.004	0.006	−0.006	0.019
Work stressors → social support → mental health	0.014	0.07	−0.020	0.049
Work stressors → social well-being → self-identity → mental health	0.023	0.010	0.005	0.043
Work stressors → social support → self-identity → mental health	0.043	0.012	0.026	0.073
Work stressors → social support → social well-being → mental health	0.005	0.008	−0.010	0.022
Work stressors → social support → social well-being → self-identity → mental health	0.031	0.007	0.020	0.048
Total mediating effect	0.252	0.024	0.204	0.300
Total effect	0.367	0.030	0.303	0.420

## Discussion

### The mediating role of social support

This study supported that academic stressors directly impacted mental health, in which social support played a mediating role. Social support had a negative prediction for COVID-19 anxiety, and results showed that it was needed to release the fear and anxiety of college students caused by COVID-19 (74, 75). And it was also negatively associated with academic stressors and work stressors, playing as a protective factor for the mental issue (41, 43, 44, 46). During COVID-19, effective support from friends and family could decrease the academic stressors of disturbed face-to-face study and worry about performance or work opportunities, according to the Stress-Buffering Hypothesis (76). In contrast, lacking social support would cause greater symptom severity in mental health disorders, such as depression, anxiety, and bipolar disorder (77).

Usually, a person who could not get in touch with others or get support from others would suffer more negative emotions or moods (42, 51–53, 59). However, the effect of social support on mental health in the SEM was not significant. There were three intermediary variables included in the current SEM and only the effect of self-identity on mental health was significant. It could be inferred that social support was not a direct or important factor for mental health in this SEM and self-identity was the most important one. Many researchers discovered that identity could be enhanced by social support (78–81). Gleibs et al. (79) also found that social support contributed to the construction of identity. That was consistent with Haslam et al. (82), which suggested an “upward spiral” involving identity and social support whereby social support increased social identification. It was reasonable that the social support enhanced self-identity, weakening its own effect on mental health at the same time.

### The mediating role of social well-being

Social well-being was one of the outcomes of positive mental health and an important factor for it (50, 83). The stressors of the public were severe during COVID-19, and some students also were concerned about their employment (84). A high level of academic stressors might result in a poor mental state, like worrying too much about the academic career or graduation, and employment causing a low level of well-being, and further launching a negative effect on mental health in the COVID-19 environment. So, well-being could be considered as a protective role between academic stressors and mental health (85). Research about the mediating effect of well-being showed that well-being played a mediating role in the relationship between social support and mental health (64, 86). Receiving social support, such as care and attention, and being valued by others could serve as an effective buffer to challenging life events and enhance mental well-being (62). Additionally, social well-being also had a close relationship with self-identity (87). Especially, the sense of personal continuity through time was related to better well-being.

Liu et al. (88) discovered that well-being could directly influence mental health. As the impact of COVID-19 was gradually expanding, many scholars and social welfare agencies warned that the measures to defeat COVID-19, such as lockdown, would have long-lasting adverse effects on individuals' social well-being and mental health (89–91). This research showed that social well-being was significantly predicted by academic stressors and work stressors, similar to Poots and Cassidy (42), indicating that the more stressors are experienced, the worse will be the mental state and the lower sense of well-being. But the path from social well-being to mental health was not significant, different from the previous research (88), which might be due to the fact that well-being and identity had a close linkage, and some conceptual overlap was there between them (92).

### The mediating role of self-identity

This study showed that academic stressors, work stressors, social support, and social well-being had a strong correlation with self-identity, and self-identity further predicted mental health. Self-identity protected against health risks among college-aged populations (63). College students who were at a low level of self-identity would worry too much about their performance in social, resulting in social anxiety (93, 94) and poor mental health (54). Besides, self-identity was indicated as the crucial factor to gain new identities (78–81). Especially, in the situation where the public was suffering from the devastating impact of COVID-19, the self-identity helped them retain confidence and positive cognition about themselves, and was the main factor predicting mental problems (95).

It was widely known that social support could release the suffering and negative consequence generated from general stress according to the Stress-Buffering Hypothesis (76). Self-identity had important self-aspects as a stress-buffering factor, having the same effect as social support and well-being (63). This might suggest that individuals' social support and social well-being enhanced their self-identity, and further reduced negative emotions or psychological disorders. People's self-conceptions were always closely linked to their psychological states. For example, if an individual felt a failure at work or encountered challenges, they always de-emphasized the importance of hard work, so as to protect his self-evaluation. So, if identity was successfully made less central to the self, ongoing problems in the identity domain or even the loss of the identity should have a less psychological impact (96).

### The psychological mechanism between academic stressors and mental health

This study gave evidence that academic stressors paid a direct impact on mental health and an indirect impact on mental health through social support, social well-being, and self-identity during the outbreak of COVID-19. The direct effect that academic stressors on mental health were significant, but the mediation path through social support to mental health was not significant, partially conformed to hypothesis 1. Some studies discussed those variables, respectively (29, 44, 97). So, there might be other important variables in the model like self-identity, which burdened more effect from academic stressors to mental health, comparing social support and social well-being. Another recent study discussed the relationships among academic stressors, social support, and well-being (42), which found that social support mediated the relationship between academic stressors and well-being. In this study, we found that academic stressors could impact social well-being directly and also indirectly through social support, which supported hypothesis 2.

Self-identity was strongly correlated with anxiety, which was the main expression of mental health (94). Lacking identity would decrease individual well-being (98). It was widely known that social support and well-being strongly positively correlated with mental health (59, 60), reflecting that an individual who had resources of support and a good state of well-being, would have less psychological problems. Among college students, academic stressors were their main problems encountered, which had a strongly negative relationship with mental health (29). All those variables were important to mental health and had their own work. Taking them into the SEM model, the results showed that academic stressors not only could directly impact mental health, but also predict mental health through social support, social well-being, and self-identity; hypothesis 3 was partially supported. And work stressor in academic stressors was important, and they might have a similar effect as academic stressors. In sum, the results partially confirmed the hypothetical model and provided new evidence to explain the psychological mechanism of the effect of academic stressors on mental health during the outbreak of COVID-19.

### The work stressor of college students under the COVID-19

College students had lots of unique stressors during the COVID-19 pandemic, and many of them were future oriented; concerns about campus closures, delays in degree completion, and, as the economy continued to decline, worry about future job prospects (16, 86). According to this study, work stressor was the important stressor and had a great impact on mental health, in line with the previous studies (19–21). In addition, there were not enough studies about the work stressor of college students. This might be due to the real situation that college students did not have a fixed job or were not ready to get into the job market. Even though, it also existed in college students. Yang et al. (99) showed that the non-graduating students had higher work stressors than the graduating students. So, it was necessary to expand the study about work stressors among college students.

### Limitations and implications

Considering the mediation model results, self-identity was the most important factor for mental health comparing social support and social well-being. It might be an “upward spiral” involving identity and social support whereby social support increased social identification. So, in-depth research can be conducted by manipulating the social support level to verify this effect in the future. Studying the concrete relationship between social support and social well-being had empirical evidence for future studies about social support and self-identity.

In addition, this was a cross-sectional study that could not explain the development of college students' mental health and how its causes played their roles. It would be better to conduct longitudinal research on college students' mental health, so as to expand this study and get a more accurate conclusion. It was important in real life to study deeply and further. The results of this study could be the base for establishing mental health protection measures, by providing more social support to make them have less stressors and more well-being, then further increasing the self-identity of college students.

Overall, the results in this study could not be generalized to the public to some extent, due to the focused group and special study time. The focused group was college students that had their own characteristics, such as age, understudying, and sitting in the transition to adults. The context of the outbreak of COVID-19 was very different from the situation before the pandemic, causing different mental states of the college students. So, it is necessary to include more extended people in the study samples, and use the corresponding data before COVID-19 to test the generalization of the mediation results.

## Conclusion

In sum, in the context of the outbreak of the COVID-19 pandemic, social support, well-being, and self-identity could partially mediate the effect of academic stressors on mental health. Academic stressors could positively directly predict mental health, while self-identity negatively directly predicts mental health. Social support and social well-being played as mediators, which could not directly impact mental health. Work stressor was an important stressor during COVID-19, and had the same role as academic stressors in the model in this study.

## Data availability statement

The raw data supporting the conclusions of this article will be made available by the authors, without undue reservation.

## Author contributions

Study conception and design and draft manuscript preparation: PL, JY, ZhZ, ZiZ, and TL. Data collection: PL, JY, and ZhZ. Analysis and interpretation of results: PL, JY, and TL. All authors reviewed the results and approved the final version of the manuscript.

## Funding

This work was supported by the Key Project of Scientific Research Program of Tianjin Education Commission (Mental Health Education) (2020ZXXL-GX32).

## Conflict of interest

The authors declare that the research was conducted in the absence of any commercial or financial relationships that could be construed as a potential conflict of interest.

## Publisher's note

All claims expressed in this article are solely those of the authors and do not necessarily represent those of their affiliated organizations, or those of the publisher, the editors and the reviewers. Any product that may be evaluated in this article, or claim that may be made by its manufacturer, is not guaranteed or endorsed by the publisher.
